# 顶空-气相色谱法测定橄榄油中6种卤化溶剂残留

**DOI:** 10.3724/SP.J.1123.2023.08018

**Published:** 2024-04-08

**Authors:** Chunni LEI, Bo WANG, Qiang GU, Huan ZHANG, Xiaomei ZHANG, Jianke LI

**Affiliations:** 1.兰州海关技术中心, 甘肃 兰州 730010; 1. Technology Center of Lanzhou Customs, Lanzhou 730010, China; 2.张家港海关综合技术中心, 江苏 张家港 215600; 2. Comprehensive Technology Center of Zhangjiagang Customs, Zhangjiagang 215600, China; 3.陇南市祥宇油橄榄开发有限责任公司, 甘肃 陇南 746000; 3. Olive Oil of Longnan Xiang Yu Development Limited Liability Company, Longnan 746000, China

**Keywords:** 顶空-气相色谱法, 卤化溶剂残留, 橄榄油, headspace gas chromatography (HS-GC), halogenated solvent residues, olive oil

## Abstract

卤化溶剂残留量是评价橄榄油质量的重要指标之一。国家橄榄油质量标准GB/T 23347-2021中规定了橄榄油中每种卤化溶剂残留量≤0.1 mg/kg、卤化溶剂残留量总和≤0.2 mg/kg,其检验方法是国际油橄榄理事会发布的COI/T.20/Doc. No 8-1990标准方法,该方法操作繁琐、重复性差、自动化程度低,不适于大批量橄榄油样品中卤化溶剂残留量的测定,而目前国家尚未制定橄榄油中卤化溶剂残留量测定的标准方法。该文建立了橄榄油中氯仿、四氯化碳、1,1,1-三氯乙烷、二溴氯甲烷、四氯乙烯和溴仿6种卤化溶剂残留量的顶空-气相色谱法。将橄榄油试样摇匀后,称取2.00 g(精确至0.01 g)于顶空瓶中,立即封盖待顶空-气相色谱仪分析,采用空白初榨橄榄油配制标准工作液,外标法定量。考察了顶空进样器的进样时间、平衡温度、平衡时间对6种卤化溶剂残留量检测的影响,在进样时间3 s、平衡温度90 ℃、平衡时间30 min时6种卤化溶剂的分析效果较好。结果表明:6种卤化溶剂在0.002~0.200 mg/kg范围内线性关系良好,相关系数≥0.9991,检出限为0.0003~0.0006 mg/kg,定量限为0.001~0.002 mg/kg,不同加标水平下的平均回收率为85.53%~115.93%,相对标准偏差(*n*=6)为1.11%~8.48%。该方法的6种卤化溶剂定量限显著低于COI/T.20/Doc. No 8-1990标准方法的定量限(0.02 mg/kg),且操作时间短,精密度高,准确性好,自动化程度高,适合大批量橄榄油样品中6种卤化溶剂残留量的测定分析。

## Abstract (en)

The residual amount of halogenated solvents in olive oil is an important indicator of its quality. The National Olive Oil Quality Standard GB/T 23347-2021 states that the residual amount of individual halogenated solvents in olive oil should be ≤0.1 mg/kg and that the total residual amount of halogenated solvents should be ≤0.2 mg/kg. COI/T.20/Doc. No. 8-1990, which was published by the International Olive Council, describes the standard method used for the determination of halogenated solvents in olive oil. Unfortunately, this method is cumbersome, has poor repeatability and low automation, and is unsuitable for the detection and analysis of residual halogenated solvents in large quantities of olive oil. At present, no national standard method for determining residual halogenated solvents in olive oil is available in China. Thus, developing simple, efficient, accurate, and stable methods for the determination of residual halogenated solvents in olive oil is imperative. In this paper, a method based on automatic headspace gas chromatography was established for the determination of residual halogenated solvents, namely, chloroform, carbon tetrachloride, 1,1,1-trichloroethane, dibromochloromethane, tetrachloroethylene, and bromoform, in olive oil. The samples were processed as follows. After mixing, 2.00 g (accurate to 0.01 g) of the olive oil sample was added into a 20 mL headspace injection bottle and immediately sealed for headspace gas chromatography analysis. Blank virgin olive oil was used to prepare a standard working solution and the external standard method for quantification. The solvents used in the preparation of halogenated solvent standard intermediates were investigated and methanol was selected as a replacement for *N*,*N*-dimethylacetamide to prepare a halogenated solvent standard intermediate owing to its safety. The effects of different injection times (1, 2, 3, 4, 5, 6 s), equilibration temperatures (60, 70, 80, 90, 100, 110, 120 ℃), and equilibration times (4, 5, 8, 10, 20, 30, 40 min) of the headspace sampler on the detection of the residual amounts of the six halogenated solvents were investigated. The optimal injection time and equilibration temperature were 3 s and 90 ℃, respectively. The method demonstrated good analytical performance for the six halogenated solvents when the equilibration time was 30 min. A methodological study was conducted on the optimized method, and the results showed that the six halogenated solvents exhibited good linear relationships in the range of 0.002-0.200 mg/kg, with correlation coefficients of ≥0.9991. The limits of detection (LODs) and quantification (LOQs) of 1,1,1-trichloroethane and bromoform were 0.0006 and 0.002 mg/kg, respectively. The LODs and LOQs of chloroform, carbon tetrachloride, dibromochloromethane, and tetrachloroethylene were 0.0003 and 0.001 mg/kg, respectively. The average recoveries under different spiked levels were 85.53%-115.93%, and the relative standard deviations (*n*=6) were 1.11%-8.48%. The established method was used to analyze 13 olive oil samples available in the market. Although no halogenated solvents were detected in these samples, a limited number of samples does not represent all olive oils. Hence, monitoring residual halogenated solvents in olive oil remains necessary for its safe consumption. The LOQs of the method for the six halogenated solvents were significantly lower than that of the COI/T.20/Doc. No. 8-1990 standard method (0.02 mg/kg). In addition, the developed method can be conducted under short operation times with high precision and degree of automation as well as good accuracy. Thus, the proposed method is suitable for the determination and analysis of the residues of the six halogenated solvents in large batches of olive oil samples.

初榨橄榄油是采用机械压榨等物理方式直接从油橄榄鲜果中制取的无任何添加剂的油品^[1]^,享有“植物油皇后”“液体黄金”“飘香的软黄金”之美称,其营养成分丰富、保健功能突出,被公认为绿色保健食用油,长期食用具有降低血糖、调节血脂、降胆固醇等功效^[2⇓-4]^,因而越来越受到广大消费者的喜爱。卤化溶剂残留量是评价橄榄油质量的重要指标之一。2022年5月1日国家橄榄油质量标准GB/T 23347-2021《橄榄油、油橄榄果渣油》正式实施^[1]^,其规定了橄榄油中每种卤化溶剂残留量≤0.1 mg/kg,卤化溶剂残留量总和≤0.2 mg/kg。卤化溶剂是指含有溴、氯等元素的有机溶剂,这类溶剂具有一定的毒性^[5]^。卤化溶剂作为萃取溶剂可获取橄榄果渣油^[6]^,或橄榄油在生产或储存过程中被卤化溶剂污染^[7,8]^,从而导致橄榄油中残留卤化溶剂。

目前,国家尚未制定橄榄油中卤化溶剂残留量测定的标准方法,而GB/T 23347-2021^[1]^中指定的检测方法是国际油橄榄理事会1990年发布的COI/T.20/Doc. No 8-1990《橄榄油中四氯乙烯的测定 气相色谱法》标准方法^[9]^,采用手动顶空气体直接进样模式,将橄榄油样品放入密闭的顶空瓶中,在70 ℃条件下平衡60 min,使残留溶剂气化达到平衡,注射器取液上气体注入气相色谱仪中测定氯仿、四氯化碳、1,1,1-三氯乙烷、二溴氯甲烷、四氯乙烯和溴仿6种卤化溶剂,该方法存在操作繁琐、手动进样抽取气体误差大、自动化程度低、精密度和准确度不高的缺点,不适于大批量样品检测分析^[10]^。自动顶空进样器与气相色谱技术结合可以分析复杂基质中的挥发性有机物,具有快速、高效、环保、灵敏度高等特点^[11,12]^。本文采用自动顶空进样器结合气相色谱法分析橄榄油中6种卤化溶剂残留量,优化自动顶空参数,旨在为橄榄油中氯仿、四氯化碳、1,1,1-三氯乙烷、二溴氯甲烷、四氯乙烯和溴仿6种卤化溶剂残留量的测定提供一种简单、高效、准确、稳定的检测方法。

## 1 实验部分

### 1.1 仪器、试剂与材料

Clarus 600气相色谱仪,配电子捕获检测器(美国PE公司); Auto HS全自动顶空进样器(中国成都科林有限公司); BT224S电子天平(德国Sartorius公司)。

质量浓度为1000 mg/L的氯仿、四氯化碳、1,1,1-三氯乙烷、二溴氯甲烷、四氯乙烯、溴仿标准溶液(上海安谱璀世标准技术服务有限公司);甲醇(色谱纯,德国Merck公司); 20 mL的顶空瓶、聚氟乙烯瓶盖(中国成都科林有限公司)。

### 1.2 标准溶液配制和试样处理

#### 1.2.1 标准溶液配制

各移取10 μL 6种卤化溶剂标准溶液,用甲醇定容至1.0 mL,配制成10 mg/L的6种卤化溶剂混合标准中间液Ⅰ;再取100 μL的10 mg/L混合标准中间液Ⅰ用甲醇定容至1.0 mL,配制成1.0 mg/L的混合标准中间液Ⅱ。

称取2.00 g(精确到0.01 g)空白初榨橄榄油(检测分析确定不含待测卤化溶剂)6份,分别置于20 mL顶空进样瓶中,向3份空白初榨橄榄油中分别迅速加入4、10、20 μL混合标准中间液Ⅱ,另外3份分别迅速加入10、20、40 μL的混合标准中间液Ⅰ,立即封盖,得到每种卤化溶剂含量分别为0.002、0.005、0.01、0.05、0.1、0.2 mg/kg的卤化溶剂混合标准工作液。标准溶液配制过程中保持顶空进样瓶直立,并在水平桌面上做快速的圆周转动,使其充分混合,配制过程中空白初榨橄榄油不能接触密封垫,如有接触,需重新配制。

#### 1.2.2 试样处理

称取2.00 g(精确至0.01 g)混匀后的橄榄油试样,置于20 mL顶空进样瓶中,立即封盖,待分析。

### 1.3 分析条件

#### 1.3.1 顶空仪器参数

进样时间:3 s;加压-置换时间:120 s;放空时间:20 s;炉温:90 ℃;恒温时间:30 min;针温:150 ℃;传输线温度:150 ℃;载气压力:70 kPa。

#### 1.3.2 气相色谱参数

色谱柱:HP-5毛细管柱(30 m×0.32 mm×0.25 μm);检测器:电子捕获检测器;检测器温度:350 ℃;进样口温度:250 ℃;载气流量:0.6 mL/min;程序升温:初始温度40 ℃,保持1.0 min,以10 ℃/min升温至80 ℃,保持3 min;分流比:5∶1。

## 2 结果与讨论

### 2.1 实验条件优化

#### 2.1.1 卤化溶剂混合标准中间液配制溶剂的选择

GB/T 23347-2021^[1]^规定橄榄油中每种卤化溶剂残留量应≤0.1 mg/kg,所以以每种卤化溶剂含量为0.1 mg/kg的混合标准工作液来考察不同配制溶剂对卤化溶剂响应的影响。COI/T.20/Doc. No 8-1990标准方法^[9]^采用*N*,*N*-二甲基乙酰胺(DMA)溶剂配制卤化溶剂混合标准中间液,而DMA具有毒性,对检验人员健康不利^[13]^,鉴于此考虑选用更为安全的甲醇替代DMA配制卤化溶剂混合标准中间液。分别向空白初榨橄榄油中加入DMA、甲醇配制卤化溶剂混合标准中间液,使每种卤化溶剂含量为0.1 mg/kg,经测定甲醇配制的卤化溶剂混合标准工作液各组分获得的色谱峰峰面积较使用DMA时高,故选用甲醇溶剂配制卤化溶剂混合标准中间液,0.1 mg/kg的卤化溶剂混合标准工作液的色谱图如图1所示。

**图 1:**
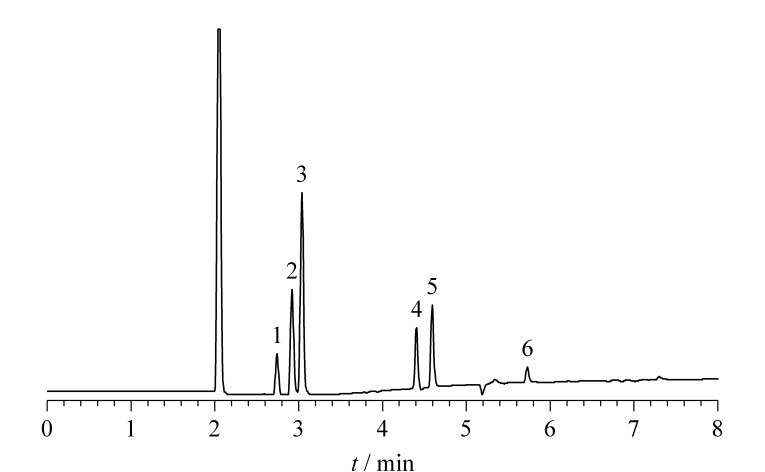
卤化溶剂混合标准工作液的色谱图

#### 2.1.2 进样时间的选择

在顶空载气压力一定的情况下,进样时间越长,进入到气相色谱仪的顶空气体体积越大,从而可提高目标物的灵敏度,但随着进样时间的延长,峰形会延展,出现色谱峰拖尾、分离不佳等现象。在平衡温度90 ℃、平衡时间30 min的条件下测定卤化溶剂混合标准工作液(每种卤化溶剂含量为0.1 mg/kg),考察不同进样时间(1、2、3、4、5、6 s)对卤化溶剂响应的影响,如图2所示。6种卤化溶剂随进样时间的延长,峰面积呈上升趋势,但随着进样时间的延长,卤化溶剂峰形逐渐变宽。在进样时间4 s和5 s时,卤化溶剂峰形变宽,导致1,1,1-三氯乙烷和氯仿色谱峰分离度较差;在进样时间6 s时,四氯化碳、1,1,1-三氯乙烷和氯仿的峰形不呈高斯分布,且1,1,1-三氯乙烷和氯仿色谱峰呈双肩峰影响定量的准确性,鉴于此选择进样时间3 s为宜。

**图 2:**
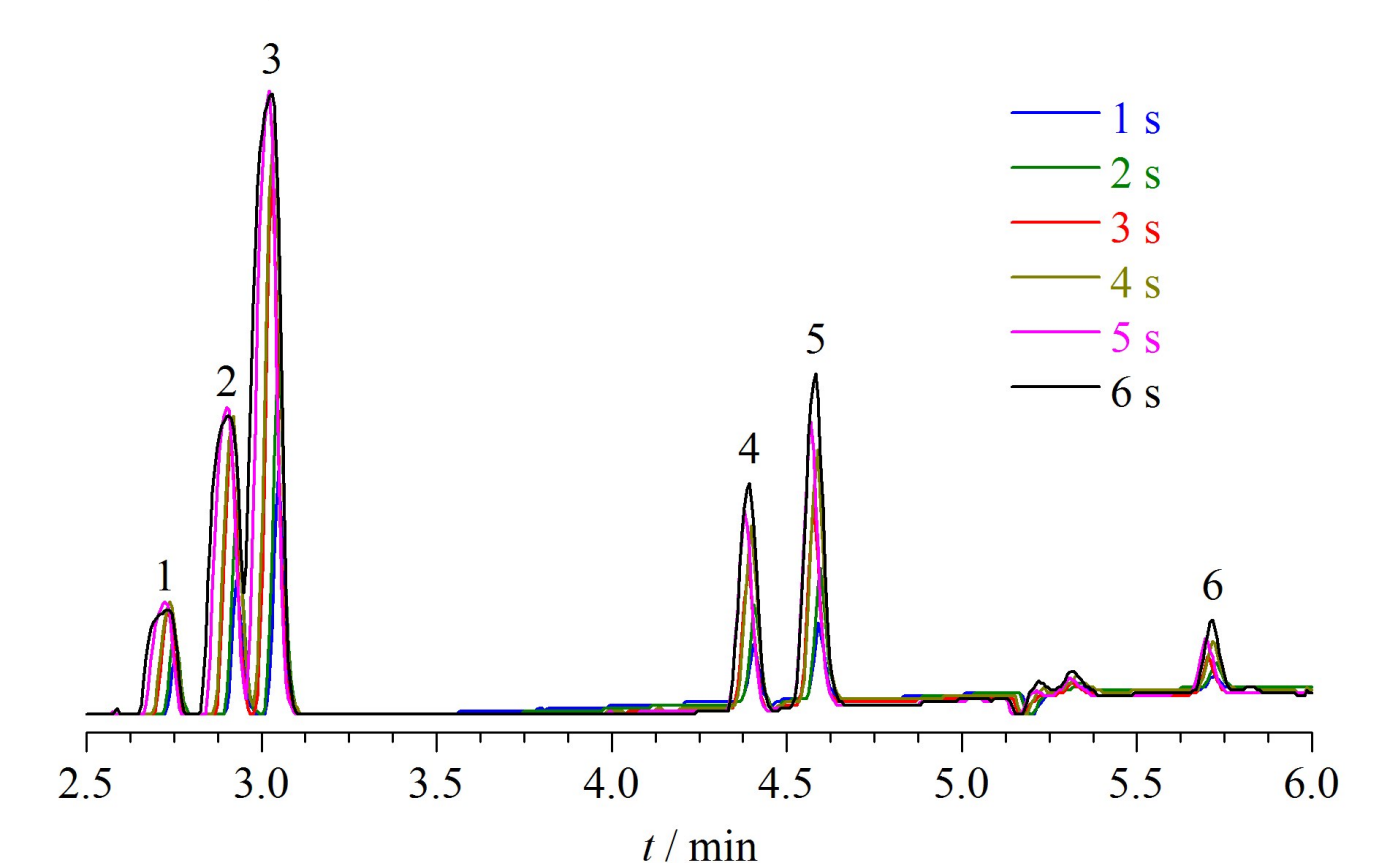
6种卤化溶剂在不同进样时间下的色谱图

#### 2.1.3 平衡温度的选择

气-液分配常数受温度影响。在一定温度范围内,目标物在气相中的平衡浓度水平随温度升高而增加^[14]^。但平衡温度过高会增加杂质峰的干扰,也有可能破坏气-液平衡的稳定性,延长分析时间^[15]^。在进样时间3 s、平衡时间30 min时考察了不同平衡温度(60、70、80、90、100、110、120 ℃)对卤化溶剂的测定效果,结果见图3。在60~120 ℃范围内,6种卤化溶剂的峰面积随温度上升而增加,表明平衡温度越高,6种卤化溶剂的顶空气体浓度越高;而温度影响的程度又因卤化溶剂组分不同而异,其中四氯化碳(沸点76.8 ℃)、1,1,1-三氯乙烷(沸点74.1 ℃)、氯仿(沸点61~62 ℃)的峰面积随平衡温度上升增加的趋势较显著,二溴氯甲烷(沸点119~120 ℃)、四氯乙烯(沸点121.2 ℃)、溴仿(沸点146~151 ℃)的峰面积随平衡温度上升增加的趋势较缓慢,说明待测组分的沸点越低,对温度影响越敏感。此外,在平衡温度≥100 ℃时,在保留时间2.588、2.647、5.348、7.324 min处检测到4个未知物峰(平衡温度110 ℃时的色谱图见图4),且随着温度升高其峰面积也随之加大,这可能是因为过高的平衡温度导致空白初榨橄榄油中的低沸点风味物质逸出,从而影响卤化溶剂的顶空动态平衡,最终对卤化溶剂的检测产生干扰。同时考虑到顶空进样器恒温状态的控制性能和顶空瓶耐压性及密封性等因素,在满足检测灵敏度要求下选择90 ℃作为最优平衡温度。

**图 3:**
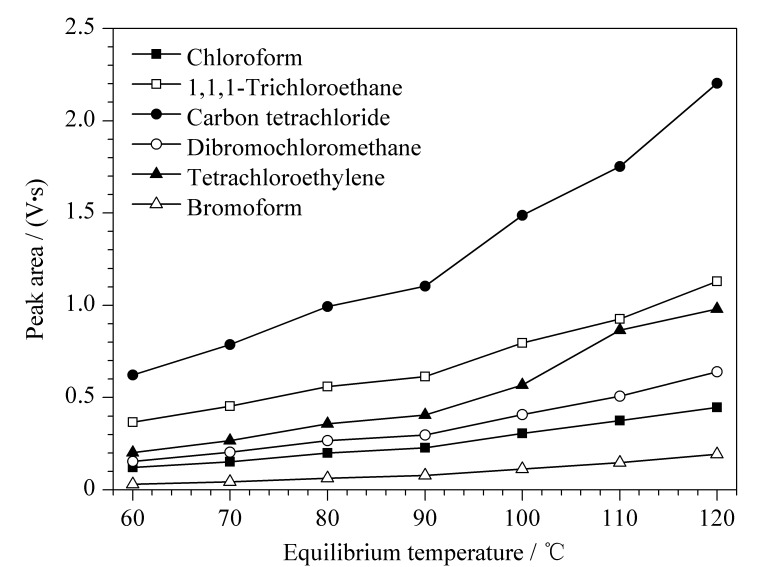
6种卤化溶剂在不同平衡温度下的峰面积

**图 4:**
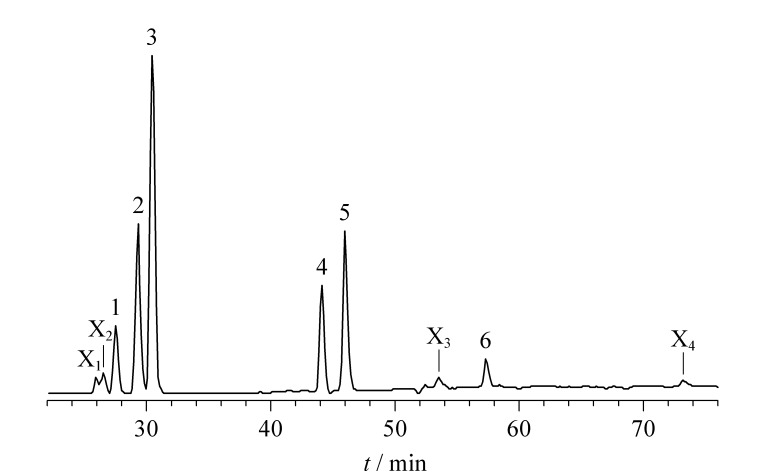
6种卤化溶剂在平衡温度110 ℃时的色谱图

#### 2.1.4 平衡时间的选择

平衡时间是卤化溶剂从橄榄油基质挥发到顶空气相中并达到气-液平衡的时间,本质上取决于被测组分分子从橄榄油基质到顶空气相的扩散速度^[16]^。平衡时间太短会导致卤化溶剂的检测灵敏度偏低,而过长的平衡时间会增加检测时长,降低检测效率。在上述优化结果的基础上,考察平衡时间(4、5、8、10、20、30、40 min)对卤化溶剂响应的影响,结果如图5所示。6种卤化溶剂达到顶空气-液平衡所需的时间不同,氯仿和溴仿在8 min时达到动态平衡,平衡时间>8 min,其峰面积未发生显著变化;1,1,1-三氯乙烷、四氯乙烯、四氯化碳、二溴氯甲烷在30 min时气-液两相达到较好平衡,再增加平衡时间,峰面积没有显著增加。综合考虑6种卤化溶剂达到气-液平衡的时间,平衡时间选择30 min。

**图 5:**
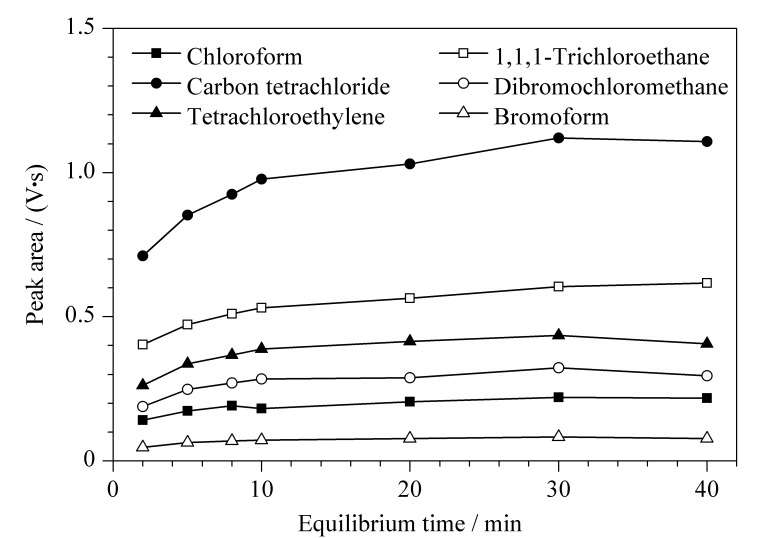
6种卤化溶剂在不同平衡时间下的峰面积

### 2.2 方法学考察

#### 2.2.1 方法的线性范围、检出限及定量限

配制好的卤化溶剂混合标准工作液按照1.3节分析条件测定,以保留时间定性,以卤化溶剂含量为横坐标(*x*, mg/kg)、峰面积(*y*)为纵坐标绘制标准曲线。由表1可知,6种卤化溶剂在0.002~0.200 mg/kg范围内线性关系良好,相关系数(*r*^2^)≥0.9991,以3倍和10倍信噪比分别计算方法的检出限(LOD)和定量限(LOQ),分别为0.0003~0.0006 mg/kg和0.001~0.002 mg/kg,该方法的卤化溶剂定量限远低于COI/T.20/Doc. No 8-1990标准方法^[9]^的定量限(0.02 mg/kg)。

**表 1:** 橄榄油基质中6种卤代溶剂的线性范围、线性方程、相关系数、检出限和定量限

Compound	Linear range/(mg/kg)	Linear equation	r^2^	LOD/(mg/kg)	LOQ/(mg/kg)
Chloroform	0.002-0.200	y=2714303x+2179	0.9991	0.0003	0.001
1,1,1-Trichloroethane	0.002-0.200	y=7453954x+12489	0.9993	0.0006	0.002
Carbon tetrachloride	0.002-0.200	y=14720065x+12436	1.0000	0.0003	0.001
Dibromochloromethane	0.002-0.200	y=3098137x+25368	0.9997	0.0003	0.001
Tetrachloroethylene	0.002-0.200	y=4544580x+19497	0.9999	0.0003	0.001
Bromoform	0.002-0.200	y=872971x+704	0.9997	0.0006	0.002

*y*: peak area; *x*: content, mg/kg.

#### 2.2.2 准确度和精密度

称取2.00 g空白初榨橄榄油,添加6种卤化溶剂混合标准中间液使其含量分别为0.002、0.005、0.010和0.100 mg/kg,每个添加水平连续测定6次,结果如表2所示。6种卤化溶剂的平均回收率为85.53%~115.93%,RSD为1.11%~8.48%(*n*=6),表明该方法的准确度和精密度较好,符合检测要求。

**表 2:** 6种卤化溶剂在不同水平下的加标回收率和 相对标准偏差(*n*=6)

Compound	Level/ (mg/kg)	Recovery/ %	RSD/ %
Chloroform	0.002	105.04	5.70
	0.005	115.93	5.76
	0.010	106.36	4.26
	0.100	109.51	2.18
1,1,1-Trichloroethane	0.002	104.64	5.04
	0.005	87.83	6.00
	0.010	105.91	3.26
	0.100	99.25	1.57
Carbon tetrachloride	0.002	103.29	5.80
	0.005	97.49	5.48
	0.010	101.04	2.46
	0.100	95.22	1.77
Dibromochloromethane	0.002	112.74	6.77
	0.005	85.53	3.18
	0.010	89.12	1.68
	0.100	93.18	1.11
Tetrachloroethylene	0.002	104.26	5.23
	0.005	92.32	5.38
	0.010	104.72	2.62
	0.100	102.99	1.39
Bromoform	0.002	105.92	8.48
	0.005	92.69	4.89
	0.010	104.03	5.30
	0.100	93.43	2.24

### 2.3 实际样品分析

随机选购13个橄榄油样品,对方法的普适性进行验证。测试的13个橄榄油样品中有8个特级初榨橄榄油、5个精炼和特级初榨混合橄榄油,测试样品均未检出6种卤化溶剂。虽然测试橄榄油样品未检测到卤化溶剂,但有限的样品数量不能代表全部橄榄油,为了食用安全,对橄榄油中卤化溶剂残留的监测仍十分必要。

## 3 结论

本文建立了顶空-气相色谱法测定橄榄油中氯仿、四氯化碳、1,1,1-三氯乙烷、二溴氯甲烷、四氯乙烯和溴仿6种卤化溶剂残留量的方法,考察了顶空进样器的进样时间、平衡温度、平衡时间对6种卤化溶剂残留量的影响,并对优化方法进行了方法学验证。建立的方法与现行有效的国际标准相比,具有一定的优越性和可替代性,其操作简单、灵敏度高、重复性好,适用于大批量橄榄油样品中卤化溶剂残留量的测定,为橄榄油安全风险评估和风险监测提供有力的技术支撑。
